# Synthesis, X-ray structure, in silico calculation, and carbonic anhydrase inhibitory properties of benzylimidazole metal complexes

**DOI:** 10.1080/14756366.2018.1481404

**Published:** 2018-07-13

**Authors:** Mehdi Bouchouit, Sofiane Bouacida, Bachir Zouchoune, Hocine Merazig, Silvia Bua, Zouhair Bouaziz, Marc Le Borgne, Claudiu T. Supuran, Abdelmalek Bouraiou

**Affiliations:** a Research Unit for Chemistry of the Environment and Molecular Structural, University of Constantine 1, Constantine, Algeria;; b Department of Materials Science, Larbi Ben Mhidi University, Oum El Bouaghi, Algeria;; c Neurofarba Department, Section of Pharmaceutical Chemistry, University of Florence, Firenze, Sesto Fiorentino (Firenze), Italy;; d Université de Lyon, Université Claude Bernard Lyon 1, Faculté de Pharmacie - ISPB, EA 4446 Bioactive Molecules and Medicinal Chemistry, SFR Santé Lyon-Est CNRS UMS3453-INSERM US7, Lyon, France

**Keywords:** Imidazole, metal complexes, X-ray crystallography, carbonic anhydrase

## Abstract

Three coordination compounds of formula {M(bmim)_2_Cl_2_} were synthetised (M = Co, Zn, and Hg) and fully characterised. Each complex incorporates 1-benzyl-2-methylimidazole (bmim) as ligand. The coordination polyhedron around the metal center for all complexes has a quasi-regular tetragonal geometry. Density functional theory calculations were carried out on the title compounds and as well on hypothetical complexes (Cu, Ni), in order to elucidate their electronic and molecular structure. The calculations reproduced the Co, Zn, and Hg experimental structures and could predict stable complexes in the case of Ni(II) and Cu(II) ions. The carbonic anhydrase (CA, EC 4.2.1.1) inhibitory effects of the three complexes were investigated. Only compound {Hg(bmim)_2_Cl_2_} (**3**) exhibited a modest inhibitory effect against hCA I, probably due to the affinity of Hg(II) for His residues at the entrance of the active site cavity.

## 1. Introduction

Metal complexes are largely investigated in chemical biology for the design of bioactive molecules useful in therapeutics such as antimicrobial^1^ and anticancer^2^ agents. Numerous metals and scaffolds can be used to get libraries of these metallodrugs with large structural diversities. Platinum opened the era of metal-based therapeutics with the well-known compound cisplatin, a leader agent in cancer treatment. More recently other complexes using cobalt, copper, and zinc were successfully employed to get therapeutics^3^^,^^4^. The organic motifs of these metallodrugs are also important to define and among a large choice of ligands, imidazole is classically used to secure the complexation of metals. For example, a recent study^5^ mentioned two new imidazole derivatives isolated from the calcareous marine sponge *Leucetta chagosensis* and identified as zinc complexes.

Classical tools (e.g. IR, UV-Vis, X-ray crystallography, elemental microanalysis) are generally used to fully characterise new complexes. Additionally quantum computational studies are precious to complete the knowledge of these metal complexes^6^. In particular, the density functional theory (DFT) using the nonlocal density approximation (LDA) BP86 functional correction is a precious tool for determining the electronic structures, the geometrical parameters, the bonding analysis, and other properties based on various calculations of organometallic and inorganic systems^7–9^. Then DFT calculations could enrich experimental studies^10^.

Taking these elements into consideration, metal complexes are currently known about their capability to inhibit human (h) carbonic anhydrase (CA, EC 4.2.1.1). For example, the inhibition activities of some cobalt(II) and zinc(II) complexes were evaluated on hCA I and hCA II^11–13^.

The aim of this study is to synthesise new metal complexes using three different metals (Co, Zn, and Hg) and the benzylimidazole as ligand. Each complex is fully investigated with the additional support of in silico calculations. An additional work was done with supplemental metals (Cu, Ni) to predict their stability using DFT calculations. Finally the three benzylimidazole metal complexes were tested as potential hCA I and hCA II inhibitors for a first biological investigation.

## 2. Materials and methods

### 2.1. Chemistry

All chemicals reagents and solvents were of analytical grade and were used as received. The 1-benzyl-2-methylimidazole (bmim) was synthesised following a literature procedure^14^ starting from 2-methylimidazole and benzyl chloride (see Supplementary Material). ^1^H-NMR and ^13^C-NMR spectra were recorded on Bruker Avance DPX250 spectrometers (Constantine, Algeria). The melting point was determined using an Electrothermal IA9100 digital melting point apparatus. UV spectra were recorded on UV/VIS Spectrophotometer Optizen 1220. IR spectra were recorded on Shimadzu FT/IR-8201 PC spectrophotometer (Constantine, Algeria).

#### 2.1.1. Preparation of {Co(bmim)_2_Cl_2_} (*1*)

A solution of 2 mmol of 1-benzyl-2-methyl-1*H*-imidazole and 1 mmol of CoCl_2_.6H_2_O in 10 ml of MeOH was stirred 24 h at room temperature. The precipitate was filtered and dried *in vacuo*. Mp 160 °C. Yield 85%. UV-Vis (chloroform, λ (nm)): 633, 614, 580, 247. IR spectrum [attenuated total reflectance (ATR)]: 3124, 2359, 1501, 1425, 1280, 1149, 1003, 727 cm^−1^. Anal. calcd. for C_22_H_24_Cl_2_CoN_4_,0.1 CHCl_3_: C 54.59, H 5.00, N 11.52; found: C 54.23, H 4.98, N 11.53.

#### 2.1.2. Preparation of {Zn(bmim)_2_Cl_2_} (*2*)

A solution of 136 mg ZnCl_2_ (1 mmol) and 344 mg (2 mmol) of 1-benzyl-2-methyl-1*H*-imidazole (L) in 10 ml of MeOH was stirred overnight at room temperature. The white precipitate **2** that formed was filtered and dried *in vacuo*. Yield 65%. Mp 163 °C. UV-Vis (chloroform, λ (nm)): 241, 553, 259, 264. IR (KBr): 3371, 3124, 3031, 2341, 2090, 1994, 1635, 1542, 1502, 1450, 1427, 1357, 1284, 1153, 1080, 1006, 756, 729, 671 cm^−1^. ^1^H NMR (250 MHz, DMSO-d_6_) δ: 7.39–7.31 (m, 3H), 7.22–7.19 (m, 2H), 6.96 (s_L_, 2H), 5.26 (s, 2H), 2.38 (s, 3H).

#### 2.1.3. Preparation of {Hg(bmim)_2_Cl_2_} (*3*)

A solution of 271 mg HgCl_2_ (1 mmol) and 344 mg (2 mmol) of 1-benzyl-2-methyl-1*H*-imidazole (L) in 10 ml of MeOH was stirred overnight at room temperature. The white precipitate **3** that formed was filtered and dried *in vacuo*. Yield 81%. Mp 140 °C. UV-Vis (chloroform, λ (nm)): 245. IR (KBr): 3406, 3128, 3028, 2368, 1959, 1878, 1797, 1620, 1539, 1496, 1434, 1353, 1280, 1195, 1149, 1076, 1002, 763, 705 cm^−1^. ^1^H NMR (250 MHz, DMSO-d_6_) δ: 6.33–6.28 (m, 3H), 6.13 (d, 2H, J = 6.09 Hz), 5.84 (s_L_, 2H), 4.17 (s, 2H), 1.30 (s, 3H).

### 2.2. X-ray crystallography studies

The crystal was coated with Paratone oil and mounted on loops for data collection. X-ray data were collected with a Bruker Apex II charge coupled device (CCD) area detector diffractometer with a graphite-monochromated Mo-Kα radiation source (0.71073 Å) at 298 K. The reported structure was solved by direct methods with SIR2002^15^ to locate all the non-H atoms, which were refined anisotropically with SHELXL97^16^ using full-matrix least squares on F^2^ procedure from within the WinGX^17^ suite of software used to prepare material for publication. All absorption corrections were performed with the SADABS program^18^. All the H atoms were placed in the calculated positions and constrained to ride on their parent atoms. Crystal data, structure refinement parameters, some intra, and intermolecular interactions hydrogen bonds, C-H…π and π–π stacking for compounds **1–3** are listed in Supplementary Material Tables S1–S3.

### 2.3. Computational methods

DFT calculations were carried out on the studied compounds using the Amsterdam Density Functional (ADF) program^19^, developed by Baerends and coworkers^20–23^. Electron correlation was treated within the LDA in the Vosko-Wilk-Nusair parametrisation^24^. The nonlocal corrections of Becke and Perdew (BP86) were added to the exchange and correlation energies, respectively^25^^,^^26^. The numerical integration procedure applied for the calculations was developed by te Velde et al^23^. The atom electronic configurations were described by a triple-ζ Slater-type orbital (STO) basis set for H 1s, C 2 s, and 2p, N 2 s and 2p, Cl 3 s and 3p augmented with a 3d single-ζ polarisation for C and N atoms and with a 2p single-ζ polarisation for H atoms. A triple-ζ STO basis set was used for the first row transition metals 3d and 4s, augmented with a 4p single-ζ polarisation function for the first row and a 6p single-ζ polarisation function for Hg. A frozen-core approximation was used to treat the core shells up to 1 s for C, N, 2p for Cl, 3p for the first row transition metals and 5p for Hg^20–24^. For the systems containing atoms in which Z > 41, the scalar relativistic zero-order regular approximation was used, with the associated optimised valence basis set. Full geometry optimisations were carried out using the analytical gradient method implemented by Versluis and Ziegler^27^. Spin-unrestricted calculations were performed for all the open-shell systems. Frequencies calculations^28^^,^^29^ were performed on all the studied compounds to check that the optimised structures are at local minima. Representation of the molecular structures and molecular orbitals were done using Amsterdam Density Functional-Graphical User Interface (ADF-GUI)^19^.

### 2.4. CA inhibition

An SX.18 MV-R Applied Photophysics (Oxford, UK) stopped-flow instrument has been used to assay the catalytic/inhibition of various CAs^30^. Phenol red (at a concentration of 0.2 mM) has been used as indicator, working at the absorbance maximum of 557 nm, with 10 mM Hepes (pH 7.5) as buffer, and 10 mM NaClO_4_ (for maintaining constant the ionic strength), following the initial rates of the CA-catalyzed CO_2_ hydration reaction for a period of 10–100 s. The CO_2_ concentrations ranged from 1.7 to 17 mM for the determination of the kinetic parameters and inhibition constants. For each inhibitor, at least six traces of the initial 5–10% of the reaction have been used for determining the initial velocity. The uncatalyzed rates were determined in the same manner and subtracted from the total observed rates. Stock solutions of inhibitor (10 mM) were prepared in dimethyl sulfoxide (DMSO) and dilutions up to 0.01 μM were done thereafter with distilled-deionised water. Inhibitor and enzyme solutions were preincubated together for 15 min at room temperature prior to assay, in order to allow for the formation of the E-I complex. The IC_50_-s were obtained by nonlinear least-squares methods using PRISM 3, as reported earlier^31–33^ and represent the mean from at least three different determinations.

## 3. Results and discussion

### 3.1. Synthesis of metal complexes

**Scheme 1.**
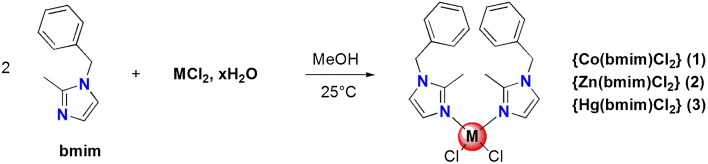
Synthesis of targeted metal complexes **1–3**.

The complexes **1–3** were prepared as depicted in Scheme 1. The ligand 1-benzyl-2-methyl-1*H*-imidazole (L) was stirred in MeOH with MCl_2_ overnight at room temperature. The solid M (II) complexes were filtered off and dried. These latter are very stable in the air. The M (II) complexes are soluble in chloroform, DMF, and DMSO but insoluble in cold methanol.

### 3.2. X-ray structural studies on metal complexes

**Figure 1.**
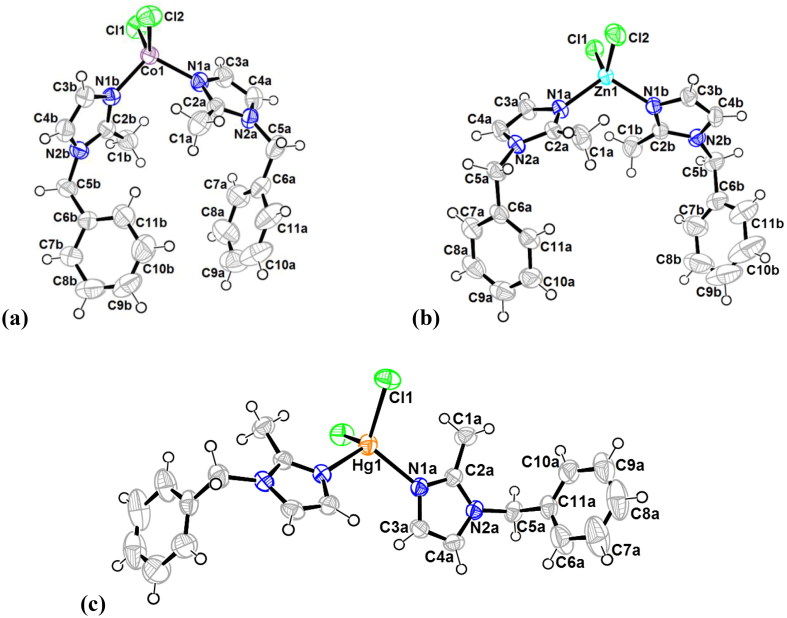
Oak ridge thermal ellipsoid plots (ORTEP) of the molecular structures of **1** (a), **2** (b) and **3** (c) in the crystal and atom numbering scheme adopted (displacement ellipsoids at the 50% probability level; H atoms with arbitrary radii; blue: nitrogen, green: chlorine).

All complexes were crystallised and suitable crystals of compounds **1**, **2,** and **3** were grown in *N*,*N*-dimethylformamide (DMF) solution of the corresponding complexes. The X-ray crystallographic analysis confirmed their respective structures and the refined X-ray crystal structures are shown in Figure 1(a–c). Crystal data and structural details of the prepared complexes are presented in Supplementary Material Table S1.

#### 3.2.1. Crystal structure of {Co(bmim)_2_Cl_2_} (*1*)

Two polymorphs of the compound {Co(bmim)_2_Cl_2_} (**1**) have been experimentally obtained and characterised. The crystal structure of the first polymorph was discussed in our previous work^34^. The second polymorph of **1**, described herein, crystallises in a monoclinic crystal system (space group P2_1_/c) and is characterised as a tetra-coordinate metal complex (Figure 1(a)). The complex contains two organic ligands of 1-benzyl-2-methylimidazole and the cobalt (II) is surrounded by two N-donor atoms and two chlorines ligands. The cobalt (II) environment exhibits a quasi-regular tetragonal coordination (Supplementary Material Table S1). The bond distances Co(1)–N(1a) and Co(1)–N(1b) are 2.016 (3) Å and 2.012 (3) Å, respectively, while the distance Co(1)–Cl(1) is 2.2378(10) Å and the distance Co(1)–Cl(2) is 2.2505(10) Å. Bond angles for N(1b)–Co(1)–Cl(2) and for Cl(1)–Co(1)–Cl(2) are 103.88 (8)° and 115.30 (4)°, respectively, and the bond angle N(1a)–Co(1)–N(2b) is 105.95(10)°. The deviation of these values from the ideal 109° corresponding to a perfect tetragonal geometry indicates a distorted tetragonal geometry.

The complex contains two organic ligands of 1-benzyl-2-methyl-1*H*-imidazole that we have labelled as molecules a and b. Each molecule consists of two rings, ring 1 with centroid Cg1: {N(1a), C(2a), N(2a), C(3a), C(4a)} and ring 2 with centroid Cg2: {C(6a), C(7a), C(8a), C(9a), C10a, C(11a)} for molecule a, and ring 3 with centroid Cg3 {N(1b), C(2b), N(2b), C(3b), C(4b)} and ring 4 with centroid Cg4: {C(6b), C(7b), C(8b), C(9b), C(10b), C(11b)} for molecule b (Figure 1(a)).

In the 1-benzyl-2-methylimidazole ligand, imidazole moiety is connected to phenyl cycle via a methylene linker. The two imidazoles (ring 1 and ring 3) are quasi-planar and form a dihedral angle with the attached corresponding phenyl cycles (ring 2 and ring 4) of 87.97(4)° and 82.38(4)°, respectively. Additional dihedral angles values between different ligand constitutional rings are summarised in Supplementary Material Table S2.

**Figure 2.**
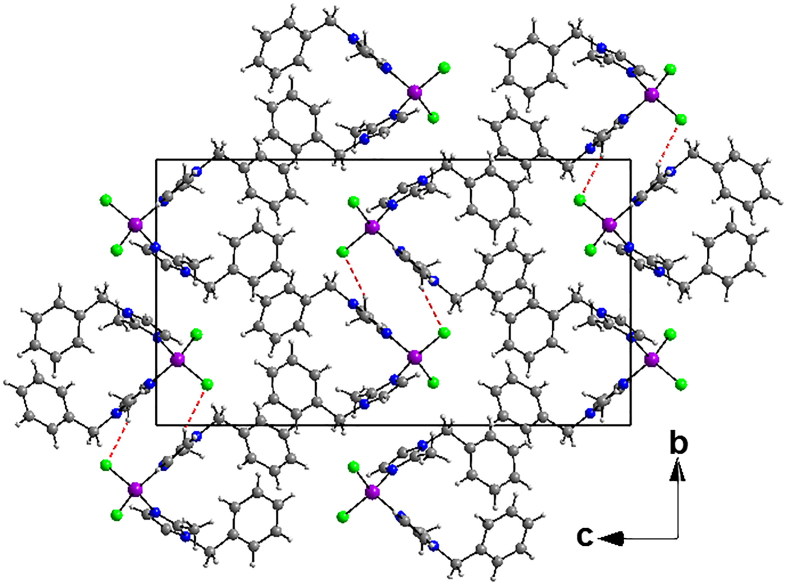
Crystal packing of **1** showing double layers in zigzag along the c axis. Hydrogen bonds are shown as red-dashed lines (C–H···Cl) connecting these layers.

The crystal packing can be described as double layers in zigzag along the c axis (Figure 2). These layers are connected with C-H…Cl hydrogen bonds. Intramolecular C-H…N hydrogen bonds interactions are also observed (Supplementary Material Table S3). The crystal structure is also supported by two strong intermolecular Cg…Cg (π–π stacking) interactions between two adjacent imidazoles with centroid (ring 1; Cg1) to centroid (ring 1; Cg1) distance of 3.547(2) Å (1-x, 1-y, 1-z) and centroid (ring 3; Cg3) to centroid (ring 3; Cg3) distance of 3.471(2) Å (-x, 2-y, 1-z). These interactions link the molecule within the layers and also link layers together and reinforcing the cohesion of the complex structure.

#### 3.2.2. Crystal structure of {Zn(bmim)_2_Cl_2_} (*2*)

The compound **2** crystallizes in a triclinic crystal system (space group P-1) (Supplementary Material Table S1, Figure 1(b)). The zinc atom is tetrahedral and surrounded by two organic ligands and two chlorine atoms. The two 1-benzyl-2-methylimidazole ligands coordinate to zinc atom as mono dentate chelating ligand. The atoms of these ligands were labeled a and b. The two chlorine atoms were joined by a covalent bond. The bonds length of Zn–N and Zn–Cl as well as the bond angles of N–Zn–Cl, N–Zn–N, and Cl–Zn–Cl are similar to those reported in complex **1** and are in the expected range.

In molecule a, the angle between ring 1 {N(1a), C(2a), N(2a), C(3a), C(4a)} and ring 2 {C(6a), C(7a), C(8a), C(9a), C(10a), C(11a)} was 65.82(6)°. By contrast, in molecule b the angle between ring 3 {N(1b), C(2b), N(2b), C(3b), C(4b)} and ring 4 {C(6b), C(7b), C(8b), C(9b), C(10b), C(11b)} was 66.56(8)°. The angle formed between the rings 2 and 4 was measured at 26.89(9)°.

**Figure 3.**
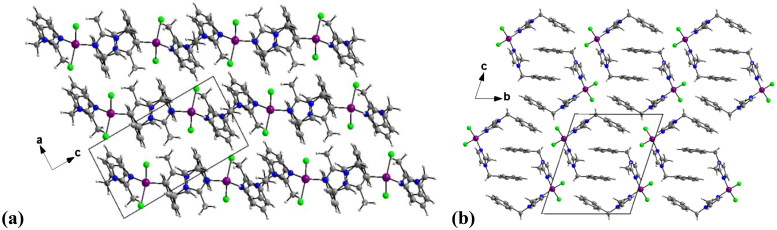
(a) View of the crystal structure of **1** in a projection along b axis showing alternating layers parallel to (101) plane. (b) Packing diagram of **2** viewed along the a axis.

The crystal packing can be described as alternating layers along the b axis parallel to (101) plane (Figure 3(a)). In these layers, each molecule is arranged as U-shape that overlaps into another one around an inversion centre (Figure 3(b)). This induces a weak π–π intermolecular interactions where the distances are 3.6041(12) Å for Cg1···Cg1, 3.6444(13) Å for Cg2···Cg2, 3.6513(12) Å for Cg3···Cg3, and 3.925(2) Å for Cg4···Cg4. Also the complex presents a very weak intermolecular interaction C(9b)–H(9b)···Cg3i (i = –x, 1–y, 1–z) where the distance of C···Cg is 3.7504(4) Å and the C-H···Cg angle is 151° (Supplementary Material Table S4).

#### 3.2.3. Crystal structure of {Hg(bmim)_2_Cl_2_} (*3*)

Compound **3** crystallises in the monoclinic crystal system (space group P 2/c) (Supplementary Material Table S1, Figure 1(c)). The asymmetric unit of compound **3** consists of one-half of the molecule, the other half being generated by a twofold rotation axis. The Hg (II) cation, lying on special position. It is in a distorted tetrahedral coordination environment and surrounded by two Cl atoms with Hg-Cl distance is 2.4629(7) Å. The two organic ligands are bound to the mercury atom through N atoms (Hg-*N* = 2.4629(7) Å). It should be noted that a significant difference in dihedral angles observed between rings (1–3 and 2–4) of the 1-benzyl-2-methylimidazole is observed compared with the previously described complexes **1** and **2** of this study (Supplementary Material Table S2).

**Figure 4.**
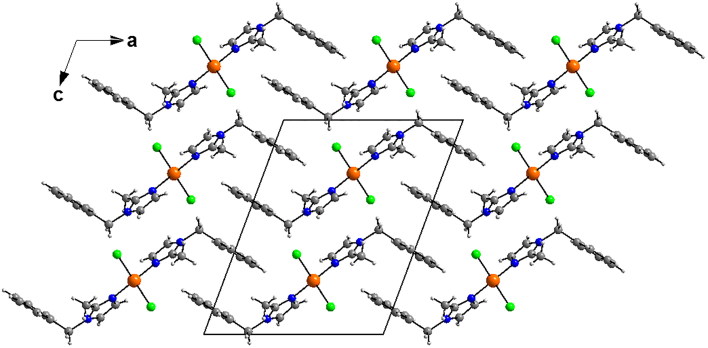
Diagram packing of **3** showing an imbricated layers parallel to (100) plane along to a axis.

The crystal packing can be described as an imbricated layers parallel to (100) plane along to a axis (Figure 4). The crystal structure is supported by weak intermolecular Cg···Cg (π–π stacking) interactions between imidazole-imidazole (Cg···Cg =3.5942(14) Å) ring and phenyl-phenyl ring (3.782(2) Å).

### 3.3. In silico calculation

**Figure 5.**
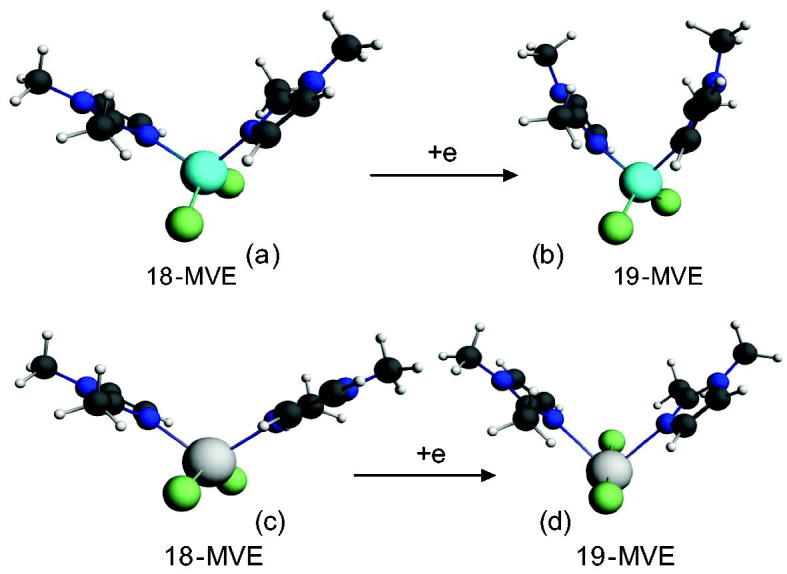
Optimised structures for (a) neutral {Zn(bmim)_2_Cl_2_} and (c) {Hg(bmim)_2_Cl_2_} and their one-electron reduced species (b) and (d), respectively.

Full geometry optimisations have been carried out on a series of characterised and hypothetical four-coordinated complexes of formula {M(bmim)_2_Cl_2_}, where benzyl rings are replaced by hydrogen atoms to reduce calculations efforts. Indeed, the characterised complexes consist of Co, Zn, and Hg metals; however, the theoretical study has been extended to hypothetical ones of Ni and Cu metals, in order to get a general overview on the bonding and on the electronic structure of the studied models according to the nature of the metal and its oxidation state. The optimised geometries are sketched in Figures 5, 8, 10, 12 and selected parameters are gathered in Supplementary Material Tables S5 and S6. The optimised geometries show that the isoelectronic neutral models of Zn and Hg (Figure 5) of singlet state (S = 0) adopt an ML_4_ tetrahedral structure around the M(II) cation as 18 metal valence electrons (MVE) species. According to the electronegativity and the metal radii, structural differences arise from the {Zn(bmim)_2_Cl_2_}and {Hg(bmim)_2_Cl_2_} models, as illustrated by the optimised geometries and geometrical parameter, the M-L bond distances are longer for the Zn model than for the Hg ones.

**Figure 6.**
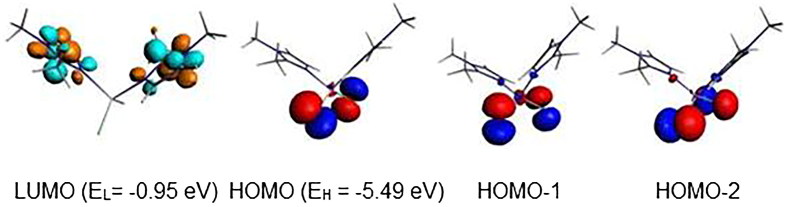
MO representations and energies of LUMO (E_L_) and HOMO (E_H_) for {Zn(bmim)_2_Cl_2_}.

**Figure 7.**
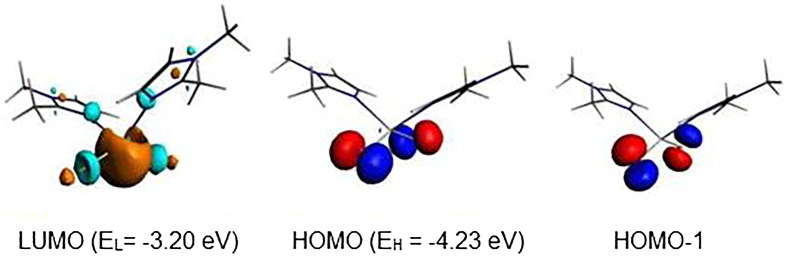
MO representations and energies of LUMO (E_L_) and HOMO (E_H_) for {Hg(bmim)_2_Cl_2_}.

The L-M-L bond angles are much open for the Hg complex than for the Zn ones. Clearly, the bond distances, the valence angles, and dihedral angles show a correspondence between the calculated and the experimental data. The geometric parameters gathered in Supplementary Material Table S5 give rise to Zn-N, Zn-Cl, Hg-N, and Hg-Cl of 2.113, 2.258, 2.347, and 2.561 Å comparable to those observed experimentally of 2.015, 2.251, 2.247, and 2.462 Å, while the N-Zn-N, Cl-Zn-Cl, N-Hg-N, and Cl-Hg-Cl bond angles of 107, 126, 114, and 132° are calculated against experimental values of 113, 117, 109, and 112°, respectively, and comparable to those reported for related complexes^35^. One can observe that the calculated bond angles around the Zn metal are comparable to those around the Hg ones, however the experimental ones are slightly close than the calculated, except for the N-Zn-N of 113° is slightly open than the calculated one of 107°. As can see from Supplementary Material Table S5, the Highest Occupied Molecular Orbital (HOMO)-Lowest Unoccupied Molecular Orbital (LUMO) gaps decrease slightly from Hg to Zn according to the following order Hg > Zn. The one electron reduction of the Zn and Hg complexes as 18-MVE species does not provoke similar modifications. Indeed, The Zn-Cl, Hg-N, and Hg-Cl bond distances undergo slight lengthening from 2.258, 2.347, and 2.561 Å to 2.285, 2.718, and 2.945 Å, respectively. In the case of the Zn-N bond, we observed a slight shortening from 2.113 to 2.080 Å, in accordance with the purely localisation on the imidazole ligand of the LUMO of Zn complex as shown in Figure 6. In parallel, the Hg-N and Hg-Cl bonds underwent significant lengthening due to the antibonding character as shown by the LUMO’s plot in Figure 7. Also, differences are emphasised on Cl-M-Cl bond angles, where those of Hg complex become much open, thus, increase from the average value of 132–172°, whereas, those of the Zn model remain almost unchanged (126 vs. 122°).

**Figure 8.**
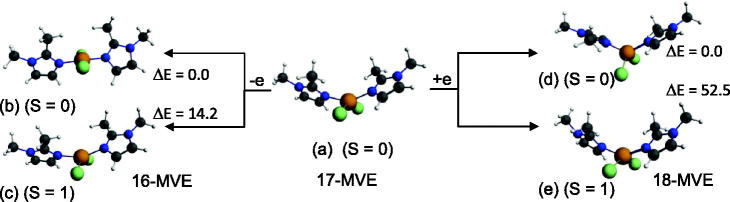
Optimised structures for (a) neutral {Cu(bmim)_2_Cl_2_}, oxidised (b) singlet and (c) triplet complexes and reduced (b) singlet and (d) triplet species. The relative energies ΔE between isomers are given in kcal/mol.

**Figure 9.**
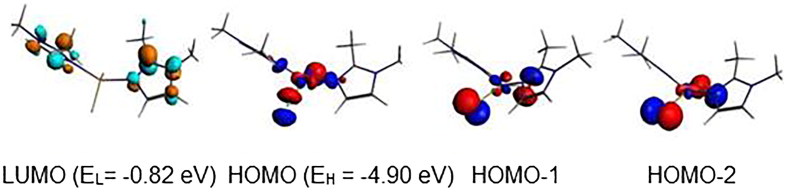
MO representations and energies of LUMO (E_L_) and HOMO (E_H_) for {Cu(bmim)_2_Cl_2_}.

The neutral {Cu(bmim)_2_Cl_2_} complex with 17-MVE is optimised in its doublet state (Figure 8), where the corresponding structure is described by an ML_4_ tetrahedral geometry around the Cu (II), but with moderate bent N-Cu-N and Cl-Cu-Cl bond angles of 145.6 and 144.5°. The one electron oxidation of the neutral {Cu(bmim)_2_Cl_2_} species caused significant shortening for {Cu(bmim)_2_Cl_2_}^+^ bond lengths for both singlet and triplet states obeying the 16-MVE configuration, where Cu-N and Cu-Cl lengths decreased from 2.207 and 2.285 Å to 1.906 and 2.188 (singlet structure, Figure 8) and 2.008 and 2.247 Å (triplet structure, Figure 8), respectively. The {Cu(bmim)_2_Cl_2_}^+^ state complex adopts square-planar geometry, which is different to that adopted by the triplet structure of tetrahedral geometry as mentioned in the Supplementary Material Table S5 and shown in Figure 8. The differences reside on the N-C-N and Cl-Cu-Cl bond angles which tend to the linearity of the singlet structure and bent in the triplet one. The singlet structure which is more stable than that of the triplet one by 14.2 kcal/mol is obtained by depopulation of the Cu-N and Cu-Cl antibonding SOMO (Figure 9). The anionic singlet structure is obtained more stable by 52.2 kcal/mol than its homolog of triplet state and exhibiting significant HOMO-LUMO gap of 1.55 eV.

**Figure 10.**
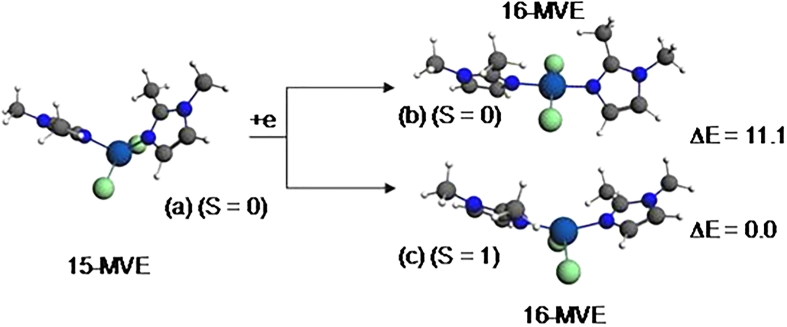
Optimised structures for the {Co(bmim)_2_Cl_2_} (a) and anionic {Co(bmim)_2_Cl_2_}^-^complexes of singlet (b) and triplet (c) states. The relative energies ΔE between isomers are given in kcal/mol.

**Figure 11.**
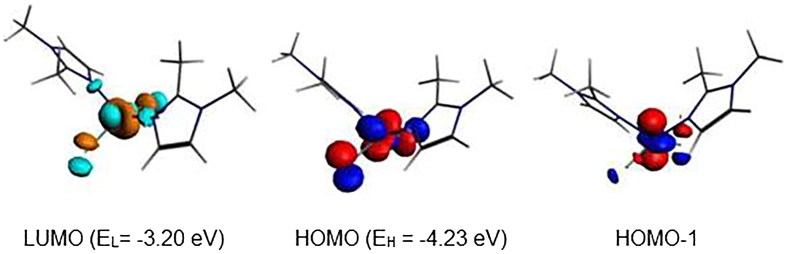
MO representations and energies of LUMO (E_L_) and SOMO (E_H_) for {Co(bmim)_2_Cl_2_}.

The optimised structure of {Co(bmim)_2_Cl_2_} complex having 15-MVE shows resemblance between the calculated parameters and the experimental ones (Figure 10), particularly those corresponding to the bond angles. As can be seen from the Supplementary Material Table S6, the average N-Co and Cl-Co bond distances of 1.989 and 2.236 are somewhat short than the experimental ones of 2.016 and 2.244 Å, respectively. The major deviation resides on the Cl-Co-Cl bond angles, where the average calculated is of 140 against the experimental value of 115.3° probably due to the crystal packing. However, the calculated dihedral angle is comparable to the experimental one. The singly occupied orbital SOMO (Figure 11) is Co-N and Co-Cl antibonding. The spin density value of 0.99 shows the localisation of the unpaired electron on the Co center. The reduced {Co(bmim)_2_Cl_2_}^–^ species of low-spin (S = 0) shows important structural modifications highlighted by the N-Co bond distance shortening (1.989 vs. 1.898 Å) and Cl-Co bond distances lengthening (2.236 vs. 2.302 Å) and N-Co-N (119 vs. 176°) and Cl-Co-Cl (140 vs. 170°) bond angles opening. It is clear that the one electron reduction of the {Co(bmim)_2_Cl_2_} neutral structure of 15-MVE adopting ML_4_ tetrahedral geometry converts into square-planar one of the monoanionic {Co(bmim)_2_Cl_2_}^-^ species of 16-MVE. It is worth noting that the low-spin is calculated less stable than that of high-spin (S = 1) by 11.1 kcal/mol inducing important structural modifications.

**Figure 12.**
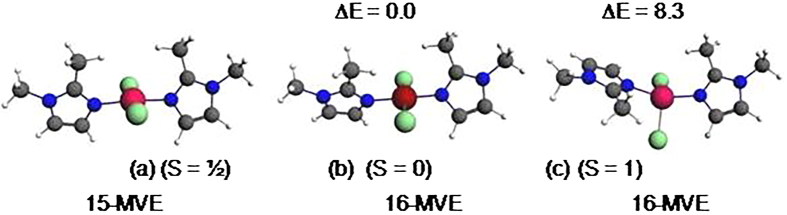
Optimised structures for {Ni(bmim)_2_Cl_2_}^+^ (a) and neutral {Ni(bmim)_2_Cl_2_} of singlet (S = 0) (b) and triplet (c) states. The relative energies ΔE between isomers are given in kcal/mol.

**Figure 13.**
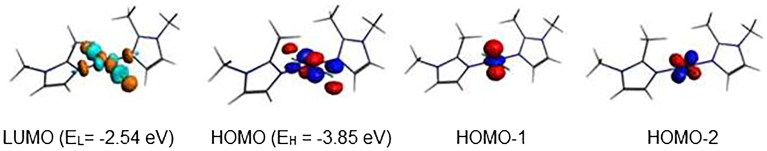
MO representations and energies of LUMO (E_L_) and HOMO (E_H_) for {Ni(bmim)_2_Cl_2_}.

The diamagnetic {Co(bmim)_2_Cl_2_}^–^ structure adopts a square-plane geometry (Figure 10, structure b), while the paramagnetic {Co(bmim)_2_Cl_2_} one having 15-MVE (Figure 10, structure c) adopts a tetrahedral geometry. However, the isoelectronic {Ni(bmim)_2_Cl_2_}^+^ species also adopts a square-planar geometry evidenced by the linear N-Ni-N and Cl-Ni-Cl bond angles of 175° (Figure 12). The {Ni(bmim)_2_Cl_2_}^+^ is obtained by one-electron oxidation of the neutral {Ni(bmim)_2_Cl_2_} which exhibits a perfect square-planar geometry and does not undergo significant geometrical modifications as clearly shown in Supplementary Material Table S6. Indeed, the low-spin neutral is computed more stable than that of triplet one (S = 1) by 8.3 kcal/mol for which the two unpaired electrons are localised on the Ni center (spin density of 1.88). The passage from the low-spin structure to high-spin one induces remarkably structural modifications concerning the Ni-N and Ni-Cl bond distances which undergo lengthening from 1.902 and 2.215 to 2.039 and 2.255, respectively, and the N-Ni-N and Cl-Ni-Cl bond angles decrease considerably from 179 and 177 to 145 and 121°, respectively, in accordance with population by one electron the LUMO which is antibonding Ni-ligands (Figure 13).

The ionisation energy and electron affinity (EA) are important parameters for the understanding the stability towards the removing of one electron from HOMO and the attachment of one electron to LUMO, respectively. Furthermore, the HOMO-LUMO gap has served as a simple measure of kinetic stability, where a molecule with a small or no HOMO-LUMO gap is chemically reactive^36^, thus, the HOMO-LUMO energy separation can be used as a simple indicator for kinetic stability, where a large gap implies high kinetic stability and low chemical reactivity, because it is energetically unfavourable to extract electrons from a low-lying HOMO and to add electrons to a high-lying LUMO. Generally in simple molecular orbital theory approaches, the HOMO energy (E_HOMO_) is related to the IP by Koopmanns’ theorem. The adiabatic ionisation potential is obtained by using Equation (1) in which M and M^+^ were the neutral and oxidised form for the optimised structures^37^. The adiabatic electron affinities are calculated by taking the difference between the total energy of the neutral ground state (M) and that of the negatively charged complexes (M^–^) for the optimised structures as given by Equation (2).

(1) $$\text{AIE }={\text{ E}}_{\text{Opt}}\left({\text{M}}^{+}\right)\;-{\text{ E}}_{\text{Opt}}\left(\text{M}\right)$$

(2) $$\text{AEA }={\text{ E}}_{\text{Opt}}\left(\text{M}\right)\;-{\text{ E}}_{\text{Opt}}\left({\text{M}}^{-}\right)$$

The negative values of –0.39 and –0.13 eV calculated for the anionic {Cu(bmim)_2_Cl_2_}^-^ and {Zn(bmim)_2_Cl_2_}^–^ of the triplet and the doublet spin states, respectively, mean that the obtained species are less stable than their neutral parents, thus, these reductions are not favourable. However, the positive values of 1.85 and 0.36 eV calculated for {Cu(bmim)_2_Cl_2_}^−^ and {Hg(bmim)_2_Cl_2_}^–^ of singlet and doublet states, respectively, mean their relative stability with regard to their corresponding neutral species. For the cobalt complex, the EA values of 0.81 and 1.91 eV show clearly the relative ease of reduction into a singlet state structure rather than into a triplet state one. The ionisation energies of 6.69, 6.75, and 7.36 eV for the obtained {Ni(bmim)_2_Cl_2_}^+^, {Cu(bmim)_2_Cl_2_}^+^ (S = 0), and {Cu(bmim)_2_Cl_2_}^+^ (S = 1) oxidised species are in accord with the HOMO’s energy (–3.85 eV) of the neutral {Ni(bmim)_2_Cl_2_} and with the SOMO’s energy of the neutral {Cu(bmim)_2_Cl_2_}.

### 3.4. Biological activity

Carbonic anhydrase inhibitory effects of the three complexes are presented in Table 1. The sulfonamide inhibitor acetazolamide (**AAZ**) was used as standard. Data reported here were obtained by a stopped flow CO_2_ hydrase assay^30^.

**Table 1.** Inhibition data of hCA I and hCA II with metal complexes **1–3**.

Compounds	IC_50_ (nM)^a^	
	hCA I	hCA II
**1**	>50,000	>50,000
**2**	30,900	>50,000
**3**	559	>50,000
**AAZ**	250	19.0

a Mean from three different assays, by a stopped flow technique (errors were in the range of ±5–10% of the reported values)^30^.

All metal complexes were weak inhibitors on both hCA isoenzymes (I and II). The metal complex incorporating Hg(II), derivative **3**, exhibited an IC_50_ value in the high nanomolar range against hCA I. The Zn(II) derivative **2** was a micromolar inhibitor of the same isoform, whereas hCA II was not significantly inhibited by these derivatives. This inhibition is probably due to the affinity of Hg(II) for His residues at the entrance of the active site cavity, which thereafter interferes with the catalytic cycle of the enzyme. The replacement of the imidazole motif by a new one will thus be necessary to obtain a second generation of metal-based derivatives with a maintained complexation of the metal ion but a markedly increased inhibitory activity on hCAs. In fact it is well known that metal complexes of heterocyclic or aromatic sulfonamides show interesting inhibitory activity against CA isoforms of human or bacterial/fungal origin^38^.

## 4. Conclusions

In summary, three coordination metal complexes were synthesised by simple method. Single crystal X-ray diffraction analysis of all complexes revealed their monomeric tetra-coordinated nature. The coordination polyhedron around the metal center may be described as a quasi-regular tetragonal geometry. By means of DFT calculations, we have investigated the electronic and molecular structures of {M(bmim)_2_Cl_2_} complexes (M = Co, Zn, Hg, Ni, Cu) for a large range of electron counts and provided a comprehensive rationalisation of the bonding within this very large family of compounds. Our calculations nicely reproduced the Co, Zn, and Hg experimental structures and could predict stable complexes in the case of Ni and Cu metals. All structures exhibited large HOMO-LUMO gaps suggesting chemical stabilities and diamagnetic behaviour. The calculated IPs and EAs are in accordance with HOMO and LUMO energies, respectively. Due the structure similarities between the prepared Co, Zn, and Hg complexes and the hypothetic Cu and Ni complexes, further investigation may need to be undertaken. The replacement of the actual imidazole moiety by another functionalised ligand (e.g. benzimidazole, azaindole) could access to a new generation of metallodrugs with a double objective: maintaining metal complexation and emergence of a real inhibitory activity on hCA. Furthermore, the predictive use of DFT calculations could help us to access to new stable complexes by choosing other metals.

## Supplementary Material

Supplemental Material
