# Supplementary material for: Synthesis, X-ray structure, in silico calculation, and carbonic anhydrase inhibitory properties of benzylimidazole metal complexes
Source: J Enzyme Inhib Med Chem. 2018 Jul 13;33(1):1150–9. doi: 10.1080/14756366.2018.1481404 (PMC6052419; doi:10.1080/14756366.2018.1481404)
Supplement: Supplemental Material [file IENZ_A_1481404_SM6051.docx]

**SUPPORTING INFORMATION**

**Synthesis, X-ray structure, in silico calculation and carbonic anhydrase inhibitory properties of benzylimidazole metal complexes**

Mehdi Bouchouit^1^, Sofiane Bouacida^1,2^, Bachir Zouchoune^1,2^, Hocine Merazig^1^, Silvia Bua^4^, Zouhair Bouaziz^3^, Marc Le Borgne^3^, Claudiu T. Supuran^4^, and Abdelmalek Bouraiou^1^

*^1^Unité de Recherche de Chimie de l’Environnement et Moléculaire Structurale, Université Frères Mentouri, Constantine 25000, Algérie, ^2^Département des sciences de la matière, Université Larbi Ben M’Hidi, Oum El Bouaghi, 04000 Oum El Bouaghi, Algérie, ^3^Université de Lyon, Université Lyon 1, Faculté de Pharmacie - ISPB, EA 4446 Bioactive Molecules and Medicinal Chemistry, SFR Santé Lyon-Est CNRS UMS3453 - INSERM US7, 69373 Lyon cedex 8, France, and ^4^Neurofarba Department, Sezione di Chimica Farmaceutica e Nutraceutica, Via U. Schiff 6, Universita Degli Studi di Firenze, Sesto Fiorentino (Firenze), Italy*

Address for correspondence: Pr. Marc Le Borgne, EA 4446 Bioactive Molecules and Medicinal Chemistry, Faculté de Pharmacie - ISPB, 8 avenue Rockefeller, 69373 Lyon cedex 8, France. Tel: +33 (0)478 777 542. Fax: +33 (0)478 777 082. E-mail: marc.le-borgne@univ-lyon1.fr

**Table of contents:**

**Chemistry.** Synthesis of 1-benzyl-2-methyl-1H-imidazole (bmim).

**Table S1.** Crystallographic data and refinement parameters for **1-3**.

**Table S2.** Dihedral angles values between different constitutional rings (molecules a and b).

**Table S3.** Distances (Å) and angles (deg) of hydrogen bond for {Co(bmim)_2_Cl_2_} **1**.

**Table S4.** Intermolecular and intramolecular interactions C–H···Cg (C–H···π; Å, deg) operating in the crystal structure of {Zn(bmim)_2_Cl_2_} **2**.

**Table S5.** Selected parameters obtained for neutral, oxidized and reduced complexes. Bond distances are given in (Å) and relative energies between isomers are given in kcal/mol. Experimental values are given in parentheses.

**Table S6.** Selected parameters obtained for neutral {Co(bmim)_2_Cl_2_} and {Ni(bmim)_2_Cl_2_}, oxidized {Ni(bmim)_2_Cl_2_}^+^ and reduced {Co(bmim)_2_Cl_2_}^-^ complexes. Experimental values are given in parentheses.

**Chemistry.** Synthesis of 1-benzyl-2-methyl-1H-imidazole (bmim).

10 mmol of 2-methyl-1*H*-imidazole were added to a suspension of potassium carbonate (10 mmol) in acetonitrile (10 mL), and then stirred for 15 min at rt. 10 mmol of benzylchloride were added to the reaction mixture and the mixture was heated at 70 °C for 72 h. After completion of the reaction, the solvent was removed and the residue was dissolved in EtOAc (20 mL), washed with H_2_O (2×10 mL) and concentrated under reduced pressure. The residue was purified by silica gel chromatography (eluent: CHCl_3_) to afford the desired product. Yield 38%. Yellow oil. UV-Vis (chloroform, λ (nm)): 290. IR (KBr): 2939, 1712, 1508, 1434, 1365, 1272, 1118, 983, 721 cm^-1^. ^1^H NMR (250.13 MHz, CDCl_3_) δ: 7.43-7.32 (m, 3H), 7.17-7.14 (m, 2H), 6.99 (d, J = 1.3 Hz, 1H), 6.84 (d, J = 1.2 Hz, 1H), 5.11 (s, 2H), 2.27 (s, 3H). ^13^C NMR (62.9 MHz, CDCl_3_) δ: 144.3, 138.0, 129.3, 128.1, 127.5, 127.1 (2 C), 120.9 (2 C), 49.2, 13.4.

**Table S1.** Crystallographic data and refinement parameters for **1-3**.

|  | **{Co(bmim)_2_Cl_2_} 1** | **{Zn(bmim)_2_Cl_2_} 2** | **{Hg(bmim)_2_Cl_2_} 3** |
| --- | --- | --- | --- |
| Formula  Formula weight  Crystal habit, color  Crystal system  Space group  a (Å)  b (Å)  c (Å)  α (º)  β (º)  γ (º)  Volume (Å^3^)  Z, Z'  Density (calculated, g cm^-3^)  Absorption coefficient (mm^-1^)  F(000)  Crystal size (mm)  θ range for data collection (º)  Reflections collected  Independent reflections  R_int_  Reflections with I ≥ 2σ(*I*)  Number of parameters  Goodness-of-fit on *F^2^*  Final R indices [I ≥ 2σ(*I*)]  R indices [all data]  Largest difference peak and hole (Å^-3^)  CCDC deposition no. | C_22_H_24_Cl_2_ N_4_Co  474.28  Prism, Blue  Monoclinic  P 2_1_/c  7.5554(2)  13.0466(5) 24.0270(9)  90  103.867(2)  90  2299.37(14)  4, 4  1.37  0.997  980  0.05×0.08×0.13  1.79 - 26.06  16233  4505  0.0518  2700  264  1.01  0.0472  *R_1_*=0.1027, *wR_2_*=0.0935  0.255, -0.240  CCDC 1441542 | C_22_H_24_Cl_2_N_4_Zn  480.72  Needle, Colorless  Triclinic  P -1  7.0986(1)  11.9495(2)  14.1520(3)  71.665(1)  86.758(1)  83.420(1)  1131.72(3)  2, 2  1.411  1.336  496  0.04×0.06×0.15  2.89 - 26.57  17134  4665  0.0259  3893  264  1.024  0.0287  *R_1_*=0.0389, *wR_2_*=0.0681  0.285, -0.225  CCDC 1441540 | C_22_H_24_Cl_2_N_4_Hg  615.94  Prism, Colorless  Monoclinic  P 2/c  11.7400(2)  6.8937(2)  14.9524(3)  90  110.449(1)  90  1133.87(4)  4, 2  1.804  7.038  596  0.09×0.11×0.13  2.85-31.61  14176  3783  0.0283  3206  133  1.024  0.0270  *R_1_*= 0.0393,  *wR_2_*= 0.0483  0.966, -1.026  CCDC 1441541 |

**Table S2.** Dihedral angles values between different constitutional rings (molecules a and b).

| **ring-ring** | Dihedral angles values (deg) | | |
| --- | --- | --- | --- |
|  | **{Co(bmim)_2_Cl_2_}** | **{Zn(bmim)_2_Cl_2_}** | **{Hg(bmim)_2_Cl_2_}** |
| **1-2** | 87.97(4) | 65.82(6) | 68.36(8) |
| **3-4** | 82.38(4) | 66.56(8) | 68.36(8) |
| **1-3** | 73.34(1) | 76.01(7) | 39.16(1) |
| **2-4** | 39.09(5) | 26.89(9) | 5.74(3) |

Ring 1: {N(1a), C(2a), N(2a), C(3a), C(4a)}; ring 2: {C(6a), C(7a), C(8a), C(9a), C10a, C(11a)}; ring 3: {N(1b), C(2b), N(2b), C(3b), C(4b)} and ring 4: {C(6b), C(7b), C(8b), C(9b), C(10b), C(11b)}.

**Table S3.** Distances (Å) and angles (deg) of hydrogen bond for {Co(bmim)_2_Cl_2_} **1**.

| D–H···A | *d*(D–H) | *d*(H···A) | *d*(D–A) | D–H–A | Symmetry |
| --- | --- | --- | --- | --- | --- |
| C(1a)–H(2)···Cl(2) | 0.96 | 2.83 | 3.700(4) | 152 | -x,1-y,1-z |
| C(11b)–H(11b) ···N(2b) | 0.93 | 2.56 | 2.884(5) | 101 | x,y,z |

**Table S4.** Intermolecular and intramolecular interactions C–H···Cg (C–H···π; Å, deg) operating in the crystal structure of {Zn(bmim)_2_Cl_2_} **2**.

| C–H…Cg | *d*(C–H) | *d*(H···Cg) | *d*(C–Cg) | C–H–Cg | Symmetry |
| --- | --- | --- | --- | --- | --- |
| C(9b)–H(9b)···Cg3 (N(1b)/C(2b)/N(2b)/C(3b)/C(4b)) | 0.93 | 2.91 | 3.750(4) | 151 | -x,1-y,1-z |

**Table S5.** Selected parameters obtained for neutral, oxidized and reduced complexes. Bond distances are given in (Å) and relative energies between isomers are given in kcal/mol. Experimental values are given in parentheses.

| Complex  Spin state | {Cu(bmim)_2_Cl_2_}^+^  S = 0 S = 1 | | {Cu(bmim)_2_Cl_2_}  S = 1/2 | {Cu(bmim)_2_Cl_2_}^-^  S = 0 S = 1 | {Zn(bmim)_2_Cl_2_} {Zn(bmim)_2_Cl_2_}^-^  S = 0 S = 1/2 | {Hg(bmim)_2_Cl_2_} {Hg(bmim)_2_Cl_2_}^-^  S = 0 S = 1/2 | |
| --- | --- | --- | --- | --- | --- | --- | --- |
| MVE | 16 | 16 | 17 | 18 18 | 18 19 | 18 | 19 |
| ΔE (kcal mol^-1^) | 0.0 | 14.2 | - | 0.0 52.5 | - - | - | - |
| Δ(H-L) (eV) | 0.66 | - | - | 1.55 - | 4.54 - | 4.62 | - |
| Average M-N | 1.906 | 2.008 | 2.207 | 2.034 2.003 | 2.113 (2.015) 2.080 | 2.347 (2.247) | 2.718 |
| Average M-Cl | 2.188 | 2.247 | 2.285 | 2.525 2.324 | 2.258 (2.251) 2.285 | 2.561 (2.462) | 2.945 |
| N-M-N (°) | 178 | 154 | 145.6 | 129 142 | 107 (113) 99 | 114 (109) | 90 |
| Cl-M-Cl (°) | 179 | 142 | 144.5 | 129 137 | 126 (117) 122 | 132 (112) | 172 |
| Metal spin density | - | - | 0.5 | - 0.51 | - 2.08 | - | - |
| Metal charge | +0.44 | +0.33 | +0.34 | 0.14 0.33 | 0.33 0.32 | 0.50 | 0.18 |
| <S^2^> | - | 2.0 | 0.75 | - 2.00 | - 0.75 | - | 0.75 |
| EA (eV) | - | - |  | 1.85 -0.39 | - -0.13 | - | 0.36 |
| IP (eV) | 6.75 | 7.36 | - | - | - - | - | - |

**Table S6.** Selected parameters obtained for neutral {Co(bmim)_2_Cl_2_} and {Ni(bmim)_2_Cl_2_}, oxidized {Ni(bmim)_2_Cl_2_}^+^ and reduced {Co(bmim)_2_Cl_2_}^-^ complexes. Experimental values are given in parentheses.

| Complex  Spin state | {Co(bmim)_2_Cl_2_} {Co(bmim)_2_Cl_2_}^-^  S = ½ S = 0 S = 1 | | {Ni(bmim)_2_Cl_2_}^+^ {Ni(bmim)_2_Cl_2_}  S = ½ S = 0 S = 1 | |
| --- | --- | --- | --- | --- |
| MVE | 15 | 16 16 | 15 | 16 16 |
| ΔE (kcal mol^-1^) | - | 11.1 0.0 | - | 0.0 8.3 |
| Δ(H-L) (eV) | - | 2.46 - | - | 1.31 - |
| Average M-N (Å) | 1.989 (2.016) | 1.898 1.996 | 1.912 | 1.902 2.039 |
| Average M-Cl (Å) | 2.236 (2.244) | 2.302 2.484 | 2.144 | 2.215 2.255 |
| N-M-N (°) | 119 (106) | 176 153 | 175 | 179 145 |
| Cl-M-Cl (°) | 140 (115) | 170 126 | 175 | 177 121 |
| Metal spin density | 0.99 | - 2.01 | 0.53 | - 1.55 |
| Metal charge | +0.09 | -0.17 -0.02 | +0.39 | +0.24 +0.36 |
| <S^2^> | 0.76 | - 2.05 | 0.76 | - 2.01 |
| AEA (eV) |  | 0.81 1.91 |  |  |
| AIE (eV) |  |  | 6.69 |  |
